# Lactaptin Induces p53-Independent Cell Death Associated with Features of Apoptosis and Autophagy and Delays Growth of Breast Cancer Cells in Mouse Xenografts

**DOI:** 10.1371/journal.pone.0093921

**Published:** 2014-04-07

**Authors:** Olga A. Koval, Anastasiya V. Tkachenko, Alexandr S. Fomin, Dmitry V. Semenov, Anna A. Nushtaeva, Elena V. Kuligina, Eugeny L. Zavjalov, Vladimir A. Richter

**Affiliations:** 1 Institute of Chemical Biology and Fundamental Medicine SB RUS, Novosibirsk, Russia; 2 Novosibirsk State University, Novosibirsk, Russia; 3 Institute of Cytology and Genetics SB RUS, Novosibirsk, Russia; Karolinska Institutet, Sweden

## Abstract

Lactaptin, the proteolytic fragment of human milk kappa-casein, induces the death of various cultured cancer cells. The mechanisms leading to cell death after lactaptin treatment have not been well characterized. In this study the *in vivo* and *in vitro* effects of a recombinant analogue of lactaptin (RL2) were examined. Following treatment with the recombinant analogue of lactaptin strong caspase -3, -7 activation was detected. As a consequence of caspase activation we observed the appearance of a sub-G1 population of cells with subdiploid DNA content. Dynamic changes in the mRNA and protein levels of apoptosis-related genes were estimated. No statistically reliable differences in *p53* mRNA level or protein level were found between control and RL2-treated cells. We observed that RL2 constitutively suppressed *bcl-2* mRNA expression and down regulated Bcl-2 protein expression in MDA-MB-231 cells. We demonstrated that RL2 penetrates cancer and non-transformed cells. Identification of the cellular targets of the lactaptin analogue revealed that α/β-tubulin and α-actinin-1 were RL2-bound proteins. As the alteration in cellular viability in response to protein stimulus can be realized not only by way of apoptosis but also by autophagy, we examined the implications of autophagy in RL2-dependent cell death. We also found that RL2 treatment induces LC3-processing, which is a hallmark of autophagy. The autophagy inhibitor chloroquine enhanced RL2 cytotoxicity to MDA-MB-231 cells, indicating the pro-survival effect of RL2-dependent autophagy. The antitumour potential of RL2 was investigated *in vivo* in mouse xenografts bearing MDA-MB-231 cells. We demonstrated that the recombinant analogue of lactaptin significantly suppressed the growth of solid tumours. Our results indicate that lactaptin could be a new molecule for the development of anticancer drugs.

## Introduction

Breast milk contains many bioactive proteins, some of which become active following partial proteolysis [1]. Lactaptin, the proteolytic fragment (residues 57–134) of human milk kappa-casein, is known to induce the death of cultured cancer cells [2], [3]. The sequence of lactaptin completely overlaps with the structure of a novel antimicrobial κ-casein peptic fragment [4]. A series of recombinant analogues of lactaptin was constructed but only one of them, RL2, containing the complete amino acid sequence of lactaptin and corresponding to 23–157 of human κ-casein, effectively induced cell death in various human and mouse tumour cells *in vitro* while having no effect on the viability of nonmalignant MSC cells [3]. The amino acids sequence of RL2 contains only one cysteine residue, which corresponds to Cys30 of human κ-casein and can be responsible for the formation of disulphide bonds [3]. We recently demonstrated that treatment of mouse hepatocarcinoma A1 cells with RL2 induced phosphatidylserine externalization, effector caspases −3, −7 activation and dissipation of mitochondrial membrane potential ΔΨ. Experiments with hepatoma-bearing mice showed that RL2 injections delay solid tumour growth and spontaneous metastases [5]. The potential for using RL2 as an anticancer drug led us to investigate the mechanism of RL2-dependent cell death in more detail.

Targeting cell death by apoptosis is the most exploited strategy in the design of anticancer drugs [6]. Apoptosis may be elicited by extrinsic (death receptor) and intrinsic (mitochondrial) molecular pathways with activation of specific proteases – the caspases [7]. Autophagy as well as apoptosis could regulate cell fate and have an influence on the outcomes of chemotherapeutic treatments of tumours. Under physiological conditions autophagy serves as one of the cellular mechanisms maintaining homeostasis by the degradation of cellular components: misfolded or aggregated proteins or damaged organelles [8]. Starvation, infection, hypoxia and other stress factors may activate autophagy. A high basal level of autophagy was shown for various cancers that increase tumour cell survival under growth-dependent hypoxia or nutrient deprivation [9]. In contrast, dysregulated autophagy may result in cell death [10]. Autophagy can be activated in response to anticancer therapy by apoptosis-inducing agents that limit drug efficacy, and blockage of autophagy can facilitate apoptosis [11], [12]. Recent studies have shown that the regulation of apoptosis and autophagy is connected and that the same regulators can control both processes [13], [14], [15]. One example is the interaction between anti-apoptotic protein Bcl-2 and autophagy-related protein Beclin 1 [16]. Bcl-2 antagonizes Bax and Bak, preventing apoptosis, whereas the interaction between Bcl-2 and Beclin 1 inhibits autophagy. Therefore the Bcl-2 level is of great significance for apoptosis and autophagy.

In this study we investigated RL2-induced cell death to identify the biomarkers associated with this process. RL2 decreased the viability of various human cancer cell lines with diverse efficiency. RL2 penetrated the cells, interacted with cytoskeleton proteins and caused apoptosis via the activation of caspases −3 and −7. RL2-dependent cell death was p53-independent and accompanied by Bcl-2 depletion. Moreover, RL2 induced autophagy-related processing of LC3-I to LC-II. The combination of RL2 with the autophagy inhibitor chloroquine (CQ) enhanced cell death in a synergistic fashion (CI  = 0.47–0.85). Experiments with mouse xenografts bearing human MDA-MB-231 adenocarcinoma cells demonstrated that the recombinant analogue of lactaptin delayed tumour growth.

## Materials and Methods

### Cell culture

Cancer cell lines MDA-MB-231, MCF-7 and SW837 were obtained from the Russian cell culture collection (Russian Branch of the ETCS, Russia, St. Petersburg). MSC cells were kindly gifted by Dr. Matveeva [3]. MCF-7 and SW837 cells were cultivated in Iscove's modified Dulbecco's media (Sigma) with 10% FBS (Gibco BRL Co., Gaithersburg, MD), 2 mM L-glutamine (Sigma), 250 mg/mL amphotericin B and 100 U/mL penicillin/streptomycin (GIBCO BRL Co., Gaithersburg, MD). MDA-MB-231 cells were cultivated in Leibovitz (L15) media (Sigma) supplemented with 10% FBS, 2 mM L-glutamine, 250 mg/ml amphotericin B and 100 U/mL penicillin/streptomycin. Cells were grown in a humidified atmosphere of 5% CO_2_ in air at 37°C and were passaged with 0.05% trypsin-EDTA every 3–4 days.

### Reagents and antibodies

The following reagents were used: Matrigel (BD Bioscience), DAPI/Antifade solution (CHEMICON International, Inc), Cell Tracker Green CMFDA (Invitrogen, Tokyo, Japan), Vybrant FAM caspase-3 and -7 assay kit (Molecular Probes), MTT (3-(4,5-dimethyl-2-thiazolyl)-2,5-diphenyl-2H-tetrazolium bromide (Sigma-Aldrich), DAPI/Antifade solution (Millipore, Temecula, CA), chloroquine (Tocris Bioscience), complete protease inhibitor cocktail (Roshe Diagnostics,Germany), PMSF (Sigma-Aldrich). Molecular weight markers were from Thermo Scientific: 14.4–116.0 kDa (#26610) and 10–250 kDa (#26619).

Antibodies against human LC3A/B, MDM2, Bax and tubulin were from Abcam, p53 and Bcl-2 were from Sigma-Aldrich, and anti-RL2 was from BioSan-R (Novosibirsk, Russia). The secondary antibodies used in this study were HRP-conjugated goat anti-mouse and goat anti-rabbit (BioSan-R, Novosibirsk, Russia) or FITC-conjugated goat anti-rabbit IgG (Sigma-Aldrich).

The recombinant analogue of lactaptin RL2 was obtained from *E.coli* and purified as described previously [3]. The 98% purity of the isolated protein was confirmed by RP-HPLC chromatography on C5 reverse phase column (Discovery BIO Wide Pore C5, Sigma) in water (0.5% TFA)-acetonitil solvent system using HPLC Station (Bio-RAD Laboratories) as well as by RP-HPLC on C18 (ProntosSIL) using Milichrom A-02 station (EcoNova, Russia).

### *In vitro* cytotoxicity assays

Cell viability in response to drug treatment was determined by MTT assay (3-(4,5-dimethyl-2-thiazolyl)-2,5-diphenyl-2H-tetrazolium bromide) as described previously [5]. Briefly, cells were seeded at 2×10^3^ cells/well in a 96-well plates in a total volume of 100 μL in IMDM medium supplemented with 2 mM L-glutamine, 100 U/mL pennicillin, 100 μg/mL streptomycin, 250 mg/mL amphotericin B and 10% FBS. Cells were incubated overnight and 100 μL of complete media containing different concentrations of RL2 was added to each well and cells were incubated for 48 hours. After incubating, MTT-solution 0.25 mg/mL was added and plates were incubated at 37°C for 4 hours. The medium was removed, followed by the addition of 0.15 mL of DMSO to each well. The plates were read at 570 and 620 nm using an Apollo LB912 plate reader (Berthold Technologies, USA). Cell viability was determined as the absorbance at A570 with reference to A620 and expressed as a means percentage of control ± SD for triplicate independent experiments.

Dose-effect data were used to calculate the combination index (CI). Standard isobologram analysis was performed using the CompuSyn software (ComboSyn, Inc., Paramus, NJ) [17].

### Measurement of DNA fragmentation

Cells were cultured at 2×10^4^ per well in six-well plates and treated with 0.15 mg/mL RL2 or PBS. After 24 h incubation cells were detached using trypsin-EDTA, fixed in 70% ethanol for 30 min and treated with 0.01% Triton X-100 and 0.5 mg/mL RNaseA (Sigma-Aldrich). For FACS analysis cells were stained in 50 μg/mL propidium iodide for 1 h. The results were analysed using the ModFit (BD Bioscience) software.

### Real-time RT-PCR

Total RNA was extracted from MDA-MB-231 cells using TRIZOL Reagent (Invitrogen, Carlsbed, CA) according to the manufacturer's instructions. Each sample for real-time RT-PCR consisted of one microgram of total RNA, 30 picomoles of primers and “Master Mix RT” (BioLabMix, Novosibirsk, Russia). Real-time RT-PCR was run on a Bio-Rad iQ5 Cycler (Hercules, CA, USA) and the data were analysed using the iQ5 system software (Bio-Rad). The mean Ct values (±SD) of three independent experiments are presented. We used the following gene-specific primers for the amplification of target genes: GAPDH1 5′-GAAGGTGAAGGTCGGAGT-3′ and GAPDH2 5′-GAAGATGGTGATGGGATTTC-3′, Bcl-2*up* 5′-GGAAACTTGACAGAGGATCATGC-3′ and Bcl-2*low* 5′-TTCTGGTGTTTCCCCCTTGG-3′, MDM2*up* 5′-GGTGCTGTAACCACCTCACA-3′ and MDM2*low* 5′-TGGCACGCCAAACAAATCTC-3′, p35*up* 5′-GGAGGAGACCATTTCCACGG-3′ and p53*low* 5′ TCACCGAGAGGTTCTGGTCT 3′.

### Measurement of protein concentration

Proteins were estimated by Bradford colorimetric assay using bovine serum albumin (Sigma) as standard [18]. Briefly, BSA (1–0.002 μg/mL) and tested protein solution (RL2 or cell lysates) were diluted in saline by 11 two-times consecutive dilutions. To each 100 μL of diluted proteins, 100 μL of Protein Assay Due Reagent (Bio-Rad Laboratories, USA) was added and 10 min later samples were read using an Apollo LB 912 plate reader (Berthold Technologies, Germany) at 595 nm.

### Conjugation of TAMRA with RL2

Succinimidyl ester of carboxy-tetramethylrhodamine was synthesized in a straightforward procedure utilizing commercially available 5(6)-carboxy-tetramethylrhodamine. The reaction mixture (150 μL), consisting of 1M *NN'*-Dicyclohexylcarbodiimide (Sigma-Aldrich), 1M *N*-Hydroxysuccinimide and 1M 5(6)-carboxy-tetramethylrhodamine (1∶1∶1, v:v:v), was incubated at 24°C for 2 h. The reaction depth was controlled by TLC on kieselgel 60F254 20×20 cm aluminum sheet (Merck, Germany) using acetonitrile/water/3-ethyl ammonium acetate (7∶2∶1) as mobile phase. The reaction product was precipitated by adding diethyl ether (5 mL) and precipitated crystals were separated by filtration and then dried on air for 10 min.

To couple protein with fluorophore we used the standard coupling method [19]. Thirty μL of dissolved in DMSO succinimidyl ester of carboxy-tetramethylrhodamine (10 mg/mL) was mixed with RL2 (0.1 mL, 4 mg/mL in 150 mM NaCl) and with 1 M NaHCO_3_ buffer, pH 9.4 (20 μL) and incubated at 24°C for 2 h. The conjugate was purified by cationic-exchange chromatography on SP-sepharose medium (GE Healthcare, Sweden) applying a solution of 0.5 M NaCl with 3 M urea and with 20 mM NaAc (pH 4.5) as mobile phase and detected at 280 nm. Then RL2-TAMRA was dialyzed against distilled water for about 24 h. The molecular weight of the conjugate was estimated by its mobility in 15% SDS-PAGE (electrode buffer 25мМ Tris-HCl pH 8,3, 250 mM glycine, 0,1% SDS) with Coomassie Blue R250 staining. Fluorescence of the conjugate in PAGE was detected using a transilluminator (Vilber Lourmat TCP-20 LC, France) at 365 nm. RL2-TAMRA concentration was estimated by Bradford assay.

### Accumulation of fluorescently labeled analogue of lactaptin in human cells

Cells (1×10^4^) were cultivated in 96-well plates to 70–80% confluent and treated with the conjugate RL2-TAMRA (0.05 mg/mL) for 1.5 hours at 37°C and 5% CO_2_. After treatment the medium was removed and cells were washed twice in PBS and fixed in ice-cold 100% methanol for 10 min. Fixed cells were treated with DAPI for 5 min, washed in PBS and analysed using the ‘IN CELL Analyzer’ system (GE Healthcare, Sweden).

### Immunocytochemistry

Cells (1×10^4^) were cultured in four-well culture slides (BD Falcon, Bedford, MA) and treated with RL2 (200 μg/mL) for 8 hours. Cells were fixed with 4% paraformaldehyde and incubated with primary antibody against LC3 (1 μg/mL) for 20 min, followed by FITC-conjugated secondary antibody (1∶1000). Primary and secondary antibodies were diluted in blocking solution (PBS supplemented with 0.05% Tween 20 and 1% gelatin). Stained cells were visualized under an Axioscop 2 PLUS fluorescence microscope (Carl Zeiss, GmbH).

### Western blot

Cells were lysed in buffer: 50 mM Tris (pH 8.0), 5 mM EDTA, 150 mM NaCl containing 0.1% SDS, 1x complete protease inhibitor cocktail and 1 mM PMSF. Seven micrograms of total protein were analysed by 15% SDS-PAGE gel and transferred to a Trance-Blot nitrocellulose membrane (Bio-RAD Laboratories, Hercules, CA) by a wet blotting procedure (100 V, 500 mA, 90 min, 15°C) using the ‘Mighty small transphor’ (GE Healthcare Bio-Science AB, USA). The membranes were saturated with BSA and incubated with primary antibodies at 4°C overnight using the following concentration: LC3 (1 μg/mL), MDM2 (2 μg/mL), Bax (1∶2000), tubulin (5 μg/mL), p53(1∶1000) and Bcl-2 (1∶1000) followed by HRP-conjugated secondary antibodies (1∶5000) using ‘SNAP i.d. 2.0’ (Millipore) protein detection system. Visualization of bound antibodies was achieved using 0.05% 4-chloro-1-naphthol in TBS (50 mM Tris, pH 8.0, 150 mM NaCl) and 0.01% hydrogen peroxide.

### The preparation of RL2-Sepharose

RL2-Separose was prepared by covalent attachment of the amino group of RL2 to ester-activated-Sepharose 4B (Sigma, # A9019) according to the manufacturer's protocol. Briefly, CH-Sepharose (250 mg) was suspended in 1 mM HCl (50 mL), filtered and washed with cold 1 mM HCl. RL2 was dissolved in 150 mM NaCl with 1 mM HCl to a final concentration 4 mg/mL and 2 mL RL2 was mixed with the resin and stirred at room temperature for 18 h. Excess of active groups were blocked with 0.1 M Tris-HCl, pH 8.0 for 1 h. To remove unbound RL2 the resin was washed sequentially by 20 mL of A: 0.1 M sodium bicarbonate; B: 0.05 M Tris-HCl, 0.5 M NaCl, pH 8.0; 0.05 M sodium acetate and C: 0.5 M NaCl, pH 4.0. Finally, resin was washed by TBS buffer, pH 7.5. Unbounded RL2 was estimated by Bradford assay. The amount of immobilized RL2 was 3 mg per 1 mL of sorbent.

### Affinity chromatography of cell lysates

MCF-7 cells (2×10^6^) were rapidly lysed in 1 mL of ice-cold TBS buffer (50 mM Tris, pH 8.0, 150 mM NaCl) containing 1% Triton-X100 and complete protease inhibitor cocktail for 10 min. Cell lysate (1 mL) was applied to the 1 mL column (RL2-Sepharose resin). Chromatography was run using the BioLogic LP low-pressure chromatography system (Bio-Rad, USA) in isocratic mode and the flow rate was 1 mL/min. After the column was washed extensively with TBS buffer, bound proteins were eluted with 300 mM NaCl, 20 mM Tris-HCl, pH 7.5; 1 M NaCl, 20 mM Tris-HCl, pH 7.5; 6 M Guanidine-HCl. Chromatography was detected at 280 nm.

### Preparation of sample for MALDI-TOF MS

Bands of interest were excised from the gel and cut into small particles (about 1 mm ×1 mm). To wash and destain the gel, 50 mM NH_4_HCO_3_ (100 μL) in 50% acetonitrile was added to the gel particles and vortex for 10 min. The solution was removed and washing was repeated three times. Gel particles were dried for 30 min in a vacuum centrifuge concentrator (Eppendorf, USA) and rehydrated in 50 mM NH_4_HCO_3_, pH 8, containing sequencing grade modified trypsin (0.02 μg/μL) (Promega, USA, # V5111) for 1 h at 4°C. Excessive solution was removed and trypsin treatment was permitted for 18 h at 37°C. To extract peptides from the gel 4-steps extraction was made with the removal of the peptide solution after each extraction. First, 25 mM NH_4_HCO_3_ (50 μL) was added and vortexed for 5 min. For the subsequent three extractions 50 μL of acetonitrile/water/formic acid (50∶45∶5, v/v/v) was added and vortexed for 5 min. The peptide solutions were pooled and dried using vacuum centrifuge concentrator. The samples were kept at −74°C until MALDI-MS was performed.

### Mass spectra recording

Mass spectra were recorded using the autoflex III MALDI-TOF mass spectrometer (Bruker Daltonics, Germany) equipped with a pulsed N_2_ laser (337 nm) in a positive reflectron mode. Ions formed by a laser beam were accelerated to 20 keV kinetic energy. The final spectra were obtained accumulating 1500 single laser shot spectrum.

The solution (50 mg/mL) of 2,5-dihydroxybenzoic acid (DHB) in aqueous acetonitrile was used as a matrix. A sample solution in 0.1% TFA in water was mixed with the same volume of matrix solution. About 1 μL of the resulting solution was deposited on the 384 ground steel target plate and allowed to dry before being introduced into the mass spectrometer. External calibration in positive mode was done using Peptide Calibration Standard II (Part No. 222570, Bruker Daltonics, Germany). Mass accuracy of about 10 ppm was usually achieved. Mass spectra were processed using FlexAnalysis 2.4 software (Bruker Daltonik GmbH, Germany). Rapid identification of proteins based on a peptide mass fingerprint search through the Swiss-Prot database was done using Mascot Server (Matrix Science Inc., USA).

### Cathepsin D activity assay

MDA-MB-231 cells (2×10^5^) were plated in six-well plates and treated with RL2 (0.2 mg/mL) or CQ (10 μM), alone or in combination, for 24 h and 48 h. Cathepsin D activity in the cell lysates was assayed using a Cathepsin D activity assay kit (Abcam, England, # ab65302). The kit is a fluorescent-based assay that utilizes the preferred Cathepsin-D substrate sequence GKPILFFRLK(Dnp)-D-R-NH2) labeled with methyl cumaryl amide (MCA). A samples preparation was made according to the manufacturer's instructions. Briefly, cells (1×10^6^) were lysed in chilled lysis buffer and incubated on ice for 10 min. Lysates were centrifuged for 5 min at 13×10^3^ rpm and supernatants were transferred to new tubes. A master mix assay (52 μL) containing reaction buffer and substrate was added to supernatants (50 μL) and incubated for 1 h. The results were analysed using a fluorometer (Cary Eclipse, Varian, Australia) at Ex/Em = 328/400 nm using 5 nm excitation and emission slits.

### *In vivo* studies

Nineteen female SCID mice (line SHO-PRKDC SCID HR/HR1EW 43375) aged 6–8 weeks were obtained from the SPF vivarium of the Institute of Cytology and Genetics SB RAS, (Novosibirsk, Russia). Mice were housed in individually ventilated cages (Animal Care Systems, Colorado, USA) in groups of one to four animals per cage with *ad libitum* food (Ssniff, Soest, Germany) and water. The mice were kept in the same room within a specific pathogen-free animal facility with a regular 14/10 hour light/dark cycles (lights on 02:00 a.m.), at a constant room temperature of 22 ± 2°C, and relative humidity approximately 45 ± 15%.

All animal experiments were carried out in compliance with the protocols and recommendations for the proper use and care of laboratory animals (ECC Directive 86/609/EEC). The protocol was approved by the Committee on the Ethics of Animal Experiments of the Administration of the Siberian Branch of the Russian Academy of Science (Permit Number: 8 - 2012).

A suspension of MDA-MB-231 cells (3.5–4×10^7^ cells/mL) in PBS was mixed with Matrigel (BD Bioscience) in a ratio of 2∶1 and 0.1 mL of suspension was injected subcutaneously into the back of each mouse. RL2 was dissolved in saline and administered intravenously (200 μL) via the tail vein when tumour size reached 100 mm^3^. The mice were randomly divided into experimental and control groups (9 and 10 animals, correspondingly) and treated intravenously three times (every second day) with RL2 (40 mg/kg) or saline (control). The tumour volumes were determined by caliper measurements every 5 days and the median tumour volume (V) was calculated as V = (*π/6 xa^2^ x b*), where *a* was the smaller of the two perpendicular tumour diameters. Eight days after the last injection, all mice were sacrificed by cervical dislocation and the tumours were excised and weighed. Antitumour activity was evaluated in terms of tumour weight.

### Statistical analysis

Student's t-test was used to compare treatment effects in cell experiments. For mouse experiments, the data are expressed as mean ± SE. The Mann-Whitney U-test was used for comparison between the two groups. A p value of less than 0.05 was considered significant.

## Results

### RL2-dependent cell death and hallmarks of apoptosis

**Figure 1:**
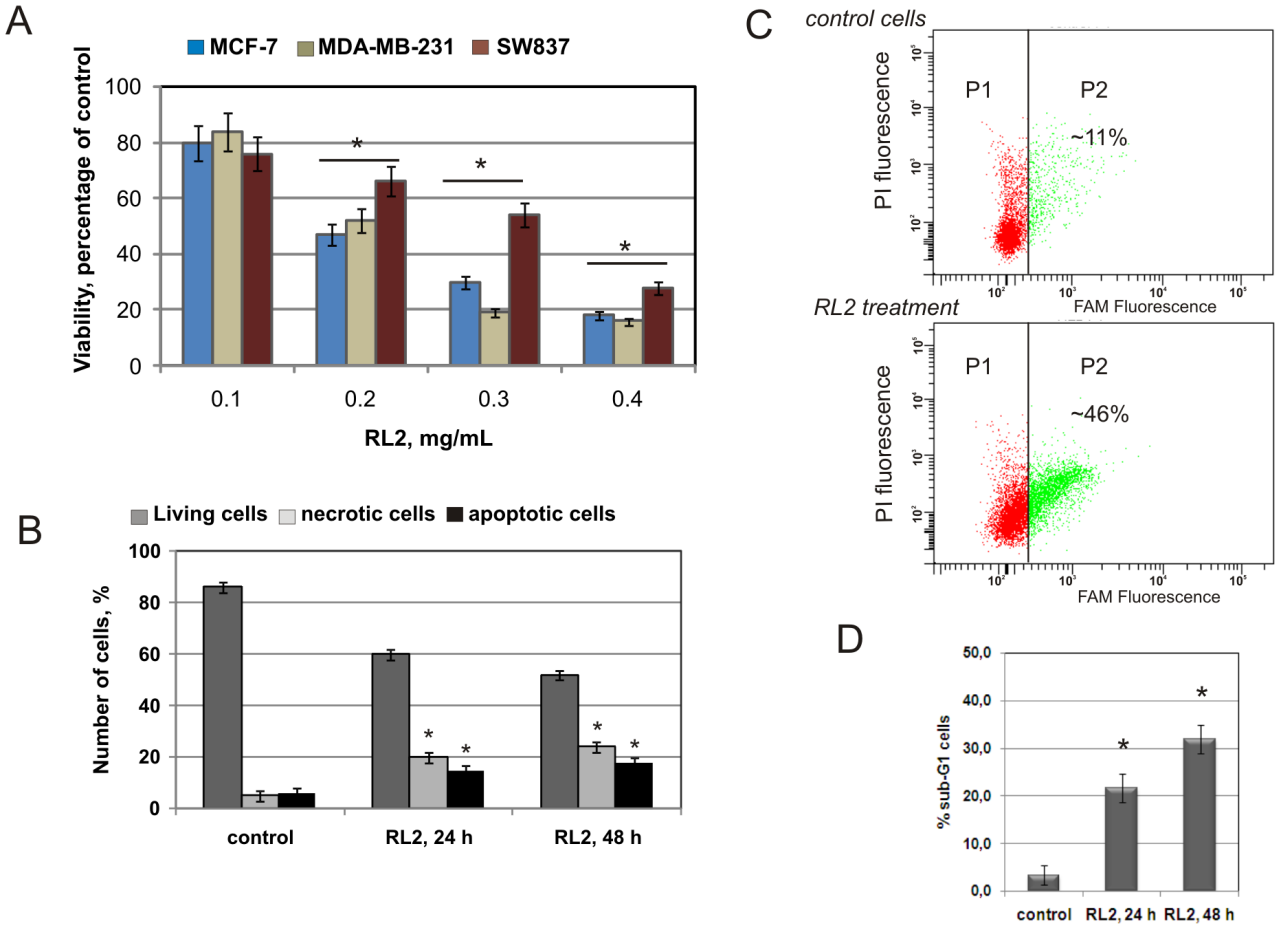
RL2 decreases viability and induces apoptosis in cultured cells. **A.** Cells were treated with different concentrations of RL2 or with saline (control) for 48 h and MTT analysis was performed. Data are presented as the mean of at least three separate experiments for each cell line. The error bars represent standard deviations (±SD). The asterisks indicate significant difference from control (*, p<0.05). **B**. MDA-MB-231 cells were incubated with RL2 (0.2 mg/mL) for 24 and 48 h then cells were stained using annexin V/propidium iodide (PI). The stained cells were assayed for apoptosis by flow cytometry. Cell populations with the annexin V^−^/PI^−^ phenotype were designated as living cells, annexin V^ +^/PI^−^ - as apoptotic cells, and annexin V^ +^/PI^+^ - as necrotic cells. Data are presented as the mean of at least four separate experiments. *, p<0.05 compared with corresponding controls. **C**. MDA-MB-231 cells were incubated with RL2 (0.2 mg/mL) for 24 h and the percentage of cells with active caspase −3, −7 were analysed using flow cytometry. FAM is fluorescein (ex 488 nm, em 530 nm). P2 population represents MDA-MB-231 cells positively stained with FAM-DEVD-FMK caspase-3 and −7 reagent. Shown are mean percentages of four independent experiments. **D**. DNA fragmentation in MDA-MB-231 cells was investigated after RL2 (0.15 mg/mL) treatment. Sub-G1 (late apoptotic) population was estimated by flow cytometry after propidium iodide staining. Data are presented as the mean of at least three separate experiments. *- significantly different from control, p<0.05.

To determine which human cancer cell line was most sensitive to the recombinant analogue of lactaptin RL2 *in vitro* we investigated two breast cancer cell lines, hormone-dependent MCF-7 and hormone-independent MDA-MB-231, and one rectum adenocarcinoma cell line, SW837. Cells were treated with RL2 (0.1 mg/mL–0.4 mg/mL) and a clear dose response was observed (Fig. 1A). Breast cancer MDA-MB-231 and MCF-7 cells showed a similar decrease in viability after RL2 treatment whereas SW837 cells were more resistant. The cells that showed the greatest sensitivity to RL2 were selected for subsequent *in vitro* experiments.

To test the nature of RL2-induced suppression of cell viability we performed annexin V/PI staining and a flow cytometry analysis of RL2-treated MDA-MB-231 cells. There was a strong decrease in the number of living cells after 24 h of incubation with RL2 (up to 60±3.4%) (Fig. 1B). The apoptotic population of annexin V-positive/PI-negative cells did not increase after 48 h. Using fluorescently labeled caspase-3 and -7 substrate and flow cytometry we quantified the percentage of cells expressing the active form of caspase-3 and -7 following treatment with the analogue of lactaptin RL2. The FLICA reagent (fluorescently labeled inhibitors of caspases) FAM-DEVD-FMK becomes covalently coupled to the active caspase-3 and -7 enzymes and the remaining green fluorescent signal is a direct measure of the amount of caspase-3 and -7 activity present in the cells [20]. Comparison of RL2-treated and untreated MDA-MB-231 cells revealed that the treatment increased P2 population containing active caspase-3 and -7 from 11±1.72% to 46±3.24% (Fig. 1C). Since DNA fragmentation is a hallmark of apoptosis we evaluated the proportion of cells containing subdiploid DNA by cell cycle analysis. As shown in Fig. 1D, RL2 treatment increased the proportion of cells with sub-G1 DNA content in a time-dependent manner: from 3.3±1.56% in the control to 21.6±2.6% and 31.8±2.84% after 24 h and 48 h of incubation, respectively.

### The influence of RL2 on the expression of key apoptosis proteins

**Figure 2:**
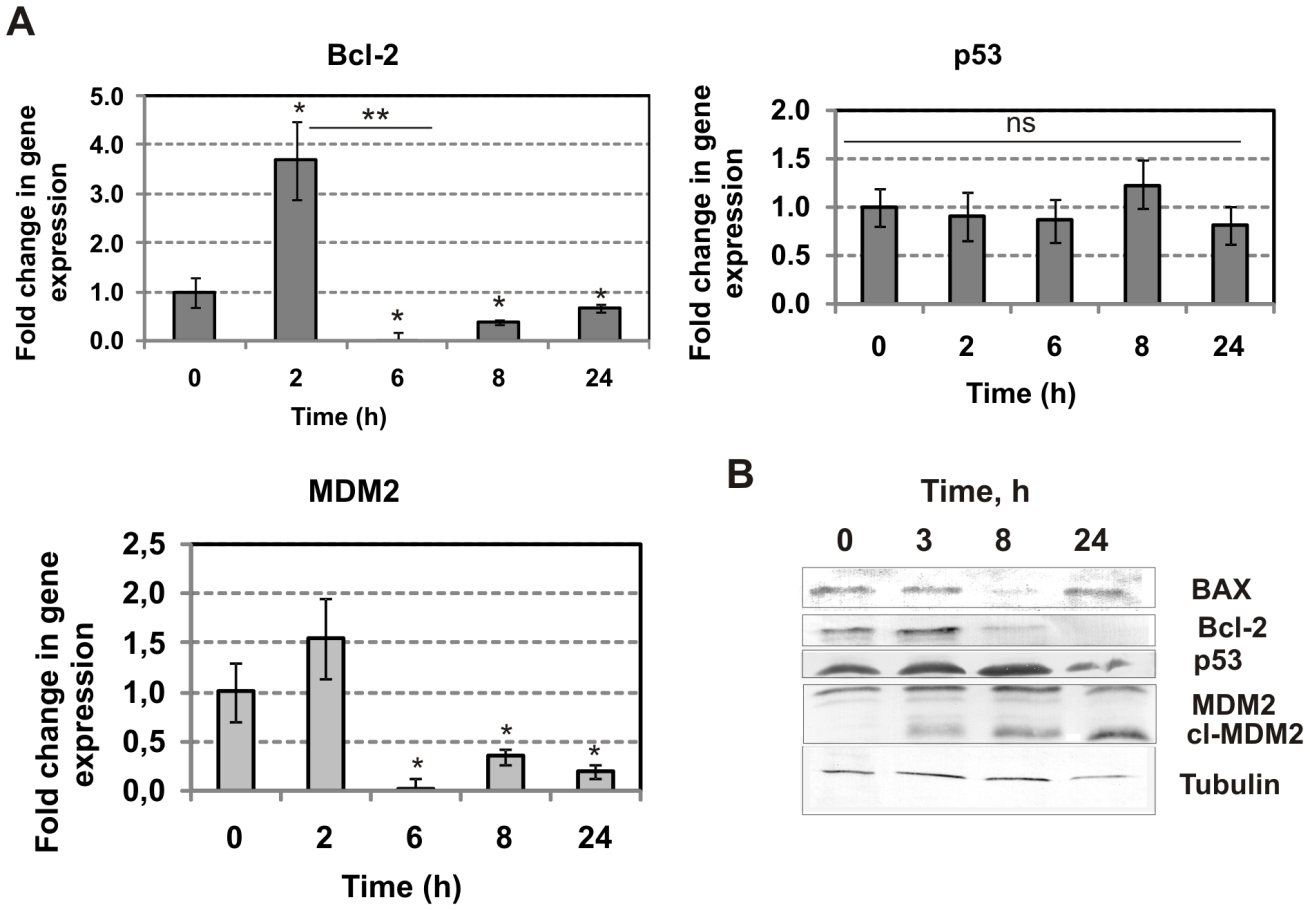
RL2 regulates activity of apoptosis-related genes. **A.** MDA-MB-231 cells were treated with RL2 (0.2 mg/mL) for the indicated time followed by total RNA isolation. Levels of transcripts were analysed by real time RT PCR with specific primers and were presented as relative values normalized to the level of GAPDH mRNA. n = 3; the error bars represent standard deviations. The asterisks indicate significant difference from control (0 h), *p<0.05, or between indicated groups, **p<0.05, ns - not significant difference between groups (p>0.05). **B**. MDA-MB-231 cells were treated with RL2 (0.2 mg/mL) followed by preparation of whole-cell lysates, which were then subjected to Western blot with the indicated antibodies. Tubulin was used as the internal control. Results shown are representative of five independent experiments.

To determine the influence of RL2 on the mRNA level of apoptosis-related genes in MDA-MB-231 cells real-time quantitative RT-PCR was performed. Dynamic changes in the mRNA level of *Bcl-2*, *p53* and *MDM2* genes were estimated. The human *GAPDH* gene was used as an internal control and the fold change in gene expression for apoptosis-related genes after RL2 treatment was determined using the delta threshold cycle (C_t_) method [21]. We found that RL2 treatment resulted in a modest induction of *Bcl-2* gene transcription after 2 h, after which the mRNA level rapidly decreased (Fig. 2A). After 24 h the Bcl-2 mRNA level in treated cells was still significantly lower relative to the mRNA level in control cells (*p*<0.05). We found that RL2 treatment caused a substantial reduction in the *mdm2* mRNA level after 6 h of incubation with no tendency to recover after 24 h. No statistically reliable change in *p53* gene transcripts was detected between control cells and RL2-treated cells during incubation (Fig. 2A).

Next we determined whether RL2-dependent changes in mRNA expression were accompanied by changes in protein expression. MDA-MB-231 cells were exposed to RL2 for 2–24 h and cellular lysates were obtained for further Western blot analysis (Fig. 2B). The expression levels of crucial apoptosis molecules, such as BAX, Bcl-2, p53 and MDM2, were analysed. Recombinant analogue of lactaptin treatment resulted in a time-dependent linear reduction in Bcl-2 level. The BAX protein in untreated cells was higher than that found at 8–24 h in RL2-treated cells. Analysis of MDM2 did not reveal any reduction in protein level but demonstrated the appearance of the cleaved form of MDM2 (*cl*-MDM2). The p53 level did not change under RL2 treatment during the period of monitoring.

### RL2 penetrates and persists in cells

**Figure 3:**
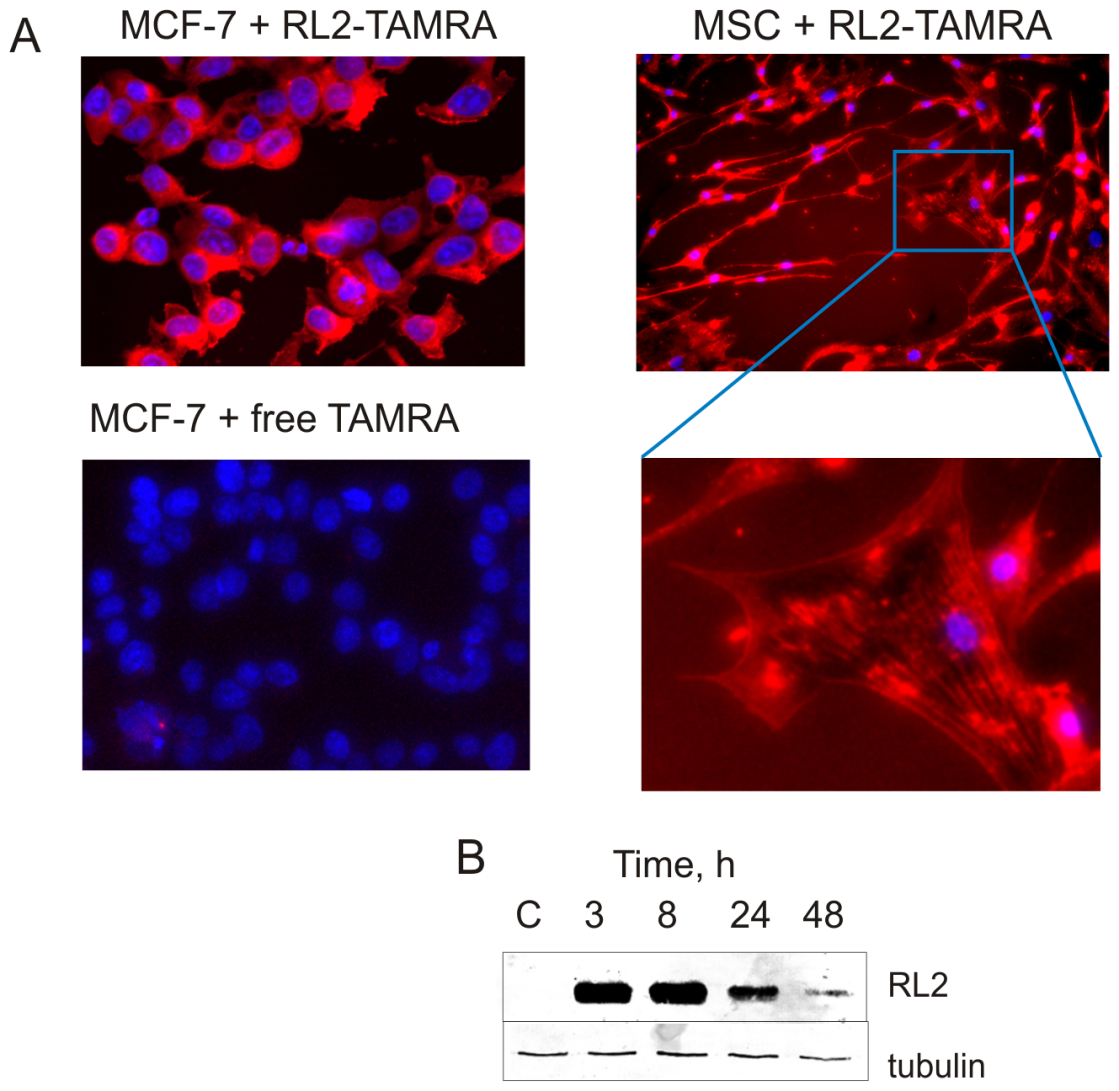
RL2 penetrates into the cells. **A.** Fluorescence microscopy analysis of MCF-7 and MSC cells was performed after treatment with RL2-TAMRA conjugate for 1.5 h using the IN CELL Analyzer system. For visualization of nuclei the cells were stained with DAPI. Images are representative of two independent experiments. **B**. Time-course of RL2 stability in MCF-7 cells. Cells were treated with RL2 (0.2 mg/mL) for the indicated time followed by the preparation of whole-cell lysates, which were then subjected to Western blot with anti-RL2 antibodies. Results shown are representative of five independent experiments.

We investigated whether RL2 is capable of penetrating into the cells or whether it interacts only with molecules on the cell's surface. For this purpose RL2 was conjugated with the fluorescent dye TAMRA, and further tumour cells (MCF-7) and nonmalignant human mesenchymal stem cells (MSC) were treated with conjugate. The IN CELL Analyser detected the intracellular internalization of RL2 conjugate (Fig. 3A). Free TAMRA did not accumulate in the cells. We observed the cytoplasmic localization of internalized RL2-TAMRA conjugate in nonmalignant MSC cells and MCF-7 cells. The efficiency of conjugate internalization was the same for the malignant and non-malignant cells. Thus RL2 did not show any specificity for penetrating tumour or non-transformed cells.

We then tested the stability of internalized RL2 by Western blot using monoclonal anti-RL2 antibodies. MCF-7 cells were incubated with RL2 (0.2 mg/mL) for 2–48 h and RL2 was detected in the cell lysates (Fig. 3B). We found RL2 in the lysates after 24 h of incubation and trace amounts of RL2 were detected even after 48 h.

### Identification of cellular target of RL2

**Figure 4:**
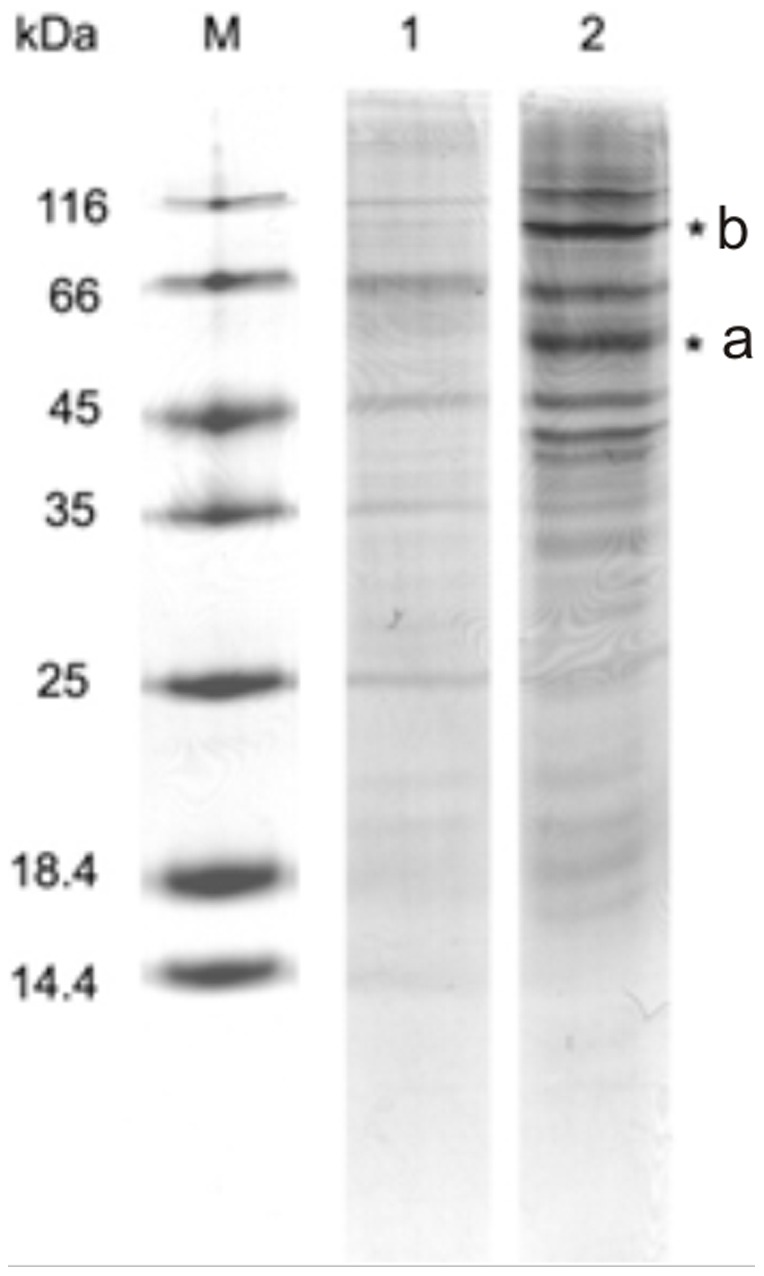
Separation of proteins of RL2-bound fraction. Coomassie stained gel. RL2-bound fractions of MCF-7 lysates were prepared as described in ‘Materials and methods’ section and then were separated by 15% SDS-PAGE gel. Bands indicated (*) were excised followed by in-gel trypsin digestion for mass spectrum analysis. Other bands were not investigated. Shown is representative gel of two independent experiments. Lane 1 – fraction eluted from non-modified Sepharose (control); Lane 2 – fraction eluted from RL2-modified Sepharose with 300 mM NaCl, flow rate was 1 mL/min and detection wavelength was 280 nm. M – molecular weights marker (14.4–116.0 kDa).

Identification of the proteins interacting with RL2 was performed by affinity chromatography of MCF-7 lysates on a column with immobilized RL2. For control of binding specificity chromatography of the lysates was also performed using non-modified Sepharose. The binding proteins were eluted using buffer solutions with a gradual increase in ionic strength. Chromatographic separation of the lysate proteins resulted in a fraction containing the RL2-bound proteins. This fraction was then analysed by 15% SDS-PAGE gel and unique bands in comparison with the control were only seen in the chromatographic fraction that was eluted by 300 mM NaCl. Two regions of RL2-bound sample had a different mobility on the gel with regard to the control (Fig. 4). They were excised for subsequent in-gel trypsin treatment. The trypsin lysates were assayed by MALDI-TOF mass-spectrometry and their spectra were analysed using FlexAnalysis 2.4 software (Table 1). Fifteen samples had a gel mobility of 45–66 kDa and 14 of these were identified as fragments of alpha- and beta- chains of human tubulin (Table 1, A). The calculated molecular masses of the α- and β-chains of tubulin were 50 kDa, which is consistent with the bands mobility in the PAGE gel. In terms of samples with a gel mobility of 66–116 kDa we found five peptides that were not present in the control samples. Using the MS-Fit program we revealed that all peptides of observed MH^+^ corresponded to human α-actinin1, the molecular mass of which is 102 kDa (Table 1, B). Thus, all RL2-interacting proteins that were identified are cytoskeletal proteins.

**Table 1:** Identification of RL2-interacting proteins A and B.

A.				
Observed [M+H]^+^, Da	Peptide	Position	Protein	Monoisotopic calculated mass, Da
1039.74	YLTVAAVFR	310–318	β-tubulin	1039.60
1085.76	EIIDLVLDR	113–121	α-tubulin	1085.62
1130.72	FPGQLNADLR	242–251	β-tubulin	1130.60
1133.63	-	-	-	
1143.77	LAVNMVPFPR	253–262	β-tubulin	1143.63
1229.72	ISEQFTAMFR	381–390	β-tubulin	1229.60
1410.89	QLFHPEQLITGK	85–96	α-tubulin	1410.77
1488.01	LISQIVSSITASLR	230–243	α-tubulin	1487.88
1620.98	LHFFMPGFAPLTSR	263–276	β-tubulin	1620.84
1702.02	AVFVDLEPTVIDEVR	65–79	α-tubulin	1701.91
1719.05	NLDIERPTYTNLNR	216–229	α-tubulin	1718.88
1757.13	IHFPLATYAPVISAEK	265–280	α-tubulin	1756.96
1959.31	GHYTEGAELVDSVLDVVR	104–121	β-tubulin	1958.98
2330.99	AFVHWYVGEGMEEGEFSEAR	403–422	α-tubulin	2330.02
2410.01	FDGALNVDLTEFQTNLVPYPR	244–264	α-tubulin	2409.21

B.				
Observed [M+H]^+^, Da	Peptide	Position	Protein	Monoisotopic calculated mass, Da
1029.80	TIPWLENR	293–300	α-actinin-1	1028.55
1215.95	LASDLLEWIR	282–291		1215.67
1387.04	VGWEQLLTTIAR	715–726		1386.77
1421.97	GYEEWLLNEIR	377–387		1421.71
2907.73	IVQTYHVNMAGTNPYTTITPQEINGK	578–603		2890.44

Protein identification was based on the SwissProt database using MALDI spectra of protein typsin lysates.

### RL2 treatment activates autophagy in MDA-MB-231 cells

**Figure 5:**
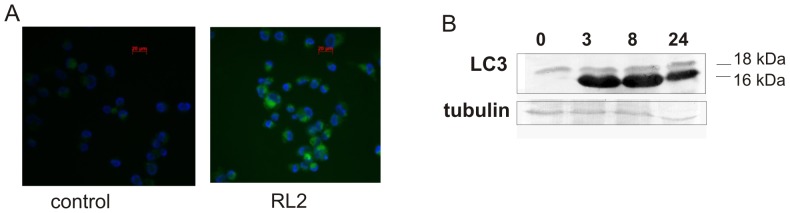
RL2 treatment induces autophagic changes in MDA-MB-231 cells. **A.** Cells were treated with RL2 (0.2 mg/mL) or non-treated (control) for 8 h and fixed with 4% paraformaldehyde followed by immunostaining with anti-LC3A/B primary and FITC-conjugated secondary antibody. Images are representative of two independent experiments. **B**. MDA-MB-231 cells were treated with RL2 (0.2 mg/mL) for various time points (0, 2, 6 and 24 h). Total lysates were prepared and subjected to Western blot analysis. Shown are representative blots of five independent experiments.

The fact that RL2 could penetrate into the cells and interact with cytoskeletal proteins led us to examine the impact of other routes of RL2-dependent cell death. Although intracellular proteins can be degraded by the autophagolysosomal pathway, we hypothesized that RL2 as well as intracellular aggregated/damaged proteins activate autophagy [22]. We examined whether the recombinant analogue of lactaptin activates autophagy in MDA-MB-231 cells. LC3 (microtubule-associated protein 1 light chain 3) is one of the autophagy-related proteins participating in the process of autophagosome formation, and is frequently used as an autophagosome marker [23], [24]. During autophagy, the cytoplasmatic LC3-I form (18 kDa) is processed to generate LC3-II (16 kDa), and this conversion could indicate autophagic activity. Here we examined the changes in LC3 during incubation with RL2. Using immunofluorescent staining we demonstrated that the population of LC3-positive cells increased after incubation of cells with RL2 (Fig. 5A). Western blot analysis indicated that the MDA-MB-231 cells treated with RL2 exhibited a significant increase in LC3-II (16 kDa) level during the initial stages of incubation (3–8 h) (Fig. 5B). Our results indicate that RL2 induces LC3 processing and may induce autophagy.

### Autophagy inhibitor CQ could enhance the efficacy of RL2

**Figure 6:**
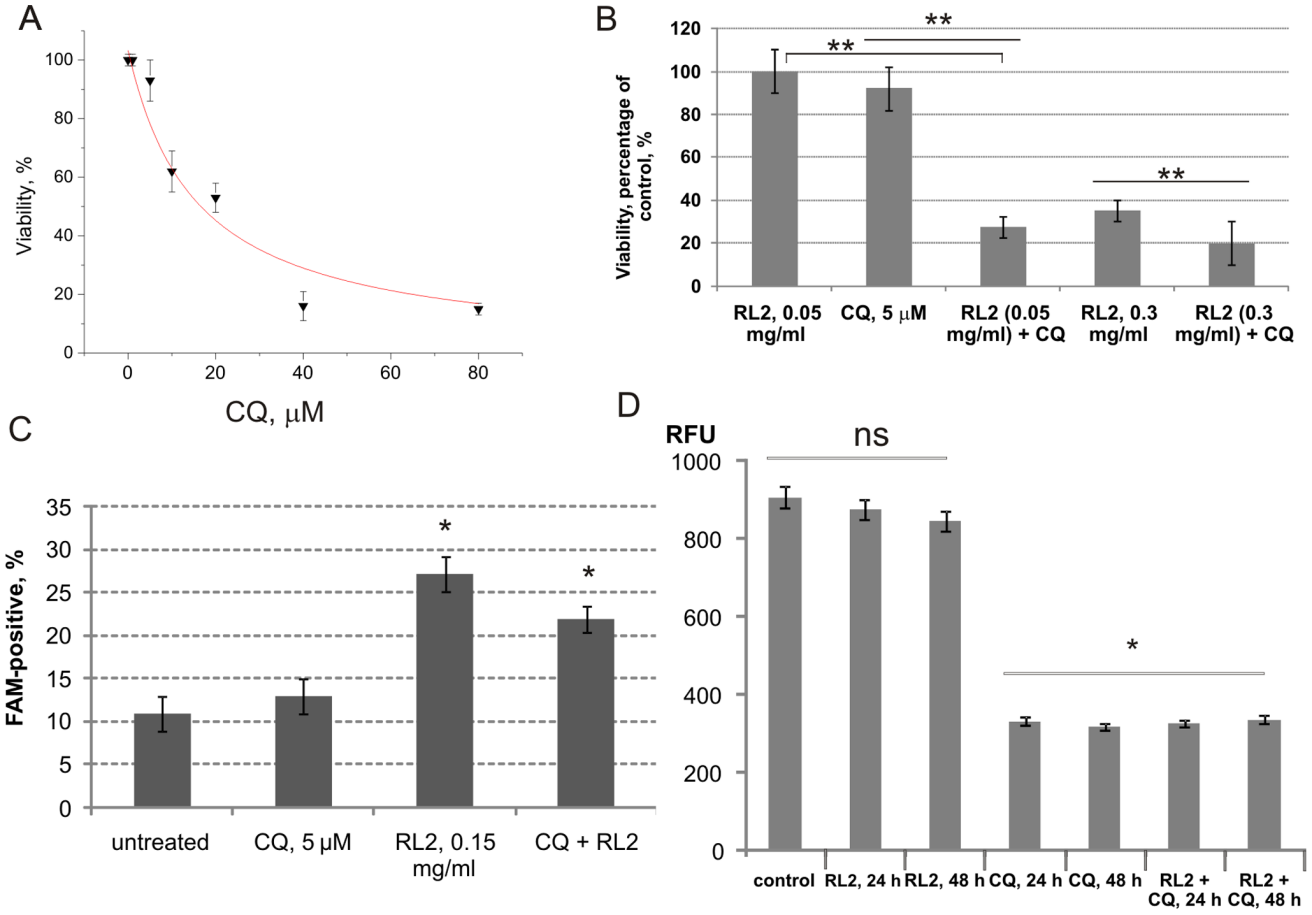
Enhanced cytotoxic outcome in combination of RL2 with CQ. MDA-MB-231 cells were used as a model. **A**. Dose-dependence of CQ cytotoxicity was calculated using MTT data of three independent experiments. Shown are mean values ± SD. **B**. Cells were treated with different concentrations of RL2 in the presence or absence of CQ, and after 48 h cell viability was measured by MTT assay. **C**. Cells were incubated with RL2 (0.2 mg/mL), CQ (5 μM) or a combination for 24 h and the percentage of cells with active caspase -3,-7 was analysed using flow cytometry. Shown are mean values ± SD of three independent experiments. The asterisks indicate significant difference from control (*, p<0.05). **D**. Cells were incubated with RL2 (0.2 mg/mL), CQ (5 μM) or a combination for 24–48 h. Cathepsin D activity was analysed using a fluorescence substrate (labeled with methyl cumaryl amide) by fluorimetry as described in ‘Materials and methods’ section. RFU – relative fluorescence units per microgram protein of sample. Shown are mean values ± SD of three independent experiments. The asterisks indicate significant difference between indicated groups (**, p<0.05), ns - not significant difference between groups (p>0.05).

Chloroquine was originally described as an antimalarial drug and was later found to inhibit autophagy in many cultured cell lines [25]. The CQ-induced death of various cell lines was associated with alteration of lysosomal function in these cells. Here we investigated the dose-dependent effects of CQ on the viability of MDA-MB-231 cells to determine which doses have a minor influence on cell viability. It was found that MDA-MB-231 cell treatment with 5 μM CQ decreased the viability of cells to 93 ± 8.2% (Fig. 6A). To assess whether inhibition of autophagy could enhance the efficacy of RL2 *in vitro* we treated MDA-MB-231 cells with a low concentration of RL2, chloroquine, or a combination of the two agents. The results of the MTT assay are presented in Fig. 6B. The combination of RL2 with CQ strongly decreased cell viability relative to either compound alone. Interactions between RL2 and CQ were determined by standard isobologram/CompuSyn software analyses [26]. It was found that a concentration of CQ as low as 5 μM in combination with RL2 (0.05–0.3 mg/mL) decreased cell viability, and the calculated combination index was CI = 0.47–0.85.

Next we tested the probable mechanisms of the observed combinatorial effect. We hypothesized that in combination each component could increase the activity of its companion: CQ could enhance caspase activation and RL2 could enhance cathepsin D activity. The activation of caspase-3 and -7 in cells treated with a combination of RL2 and CQ was estimated by flow cytometry. No quantitative changes in the cell proportion with activated caspases were seen for RL2 or the combination of RL2 and CQ (Fig. 6C) – about 27% and 22%, respectively. We can conclude that the combination of RL2 and CQ did not increase caspase activity in the treated cells.

It is known that chloroquine affects the levels and subcellular distribution of the lysosomal protease, cathepsin D [27]. We further defined the activation of cathepsin D following treatment with RL2, CQ, or the combination of these agents using a fluorometric technique. We found that RL2 did not activate cathepsin D (Fig. 6D). The combination of RL2 with CQ did not increase the activity of cathepsin D compared with CQ alone. In summary, the recombinant analogue of lactaptin and CQ synergistically enhance cell death processes by activating different types of protease.

### Recombinant analogue of lactaptin RL2 suppresses MDA-MB-231 xenograft growth

**Figure 7:**
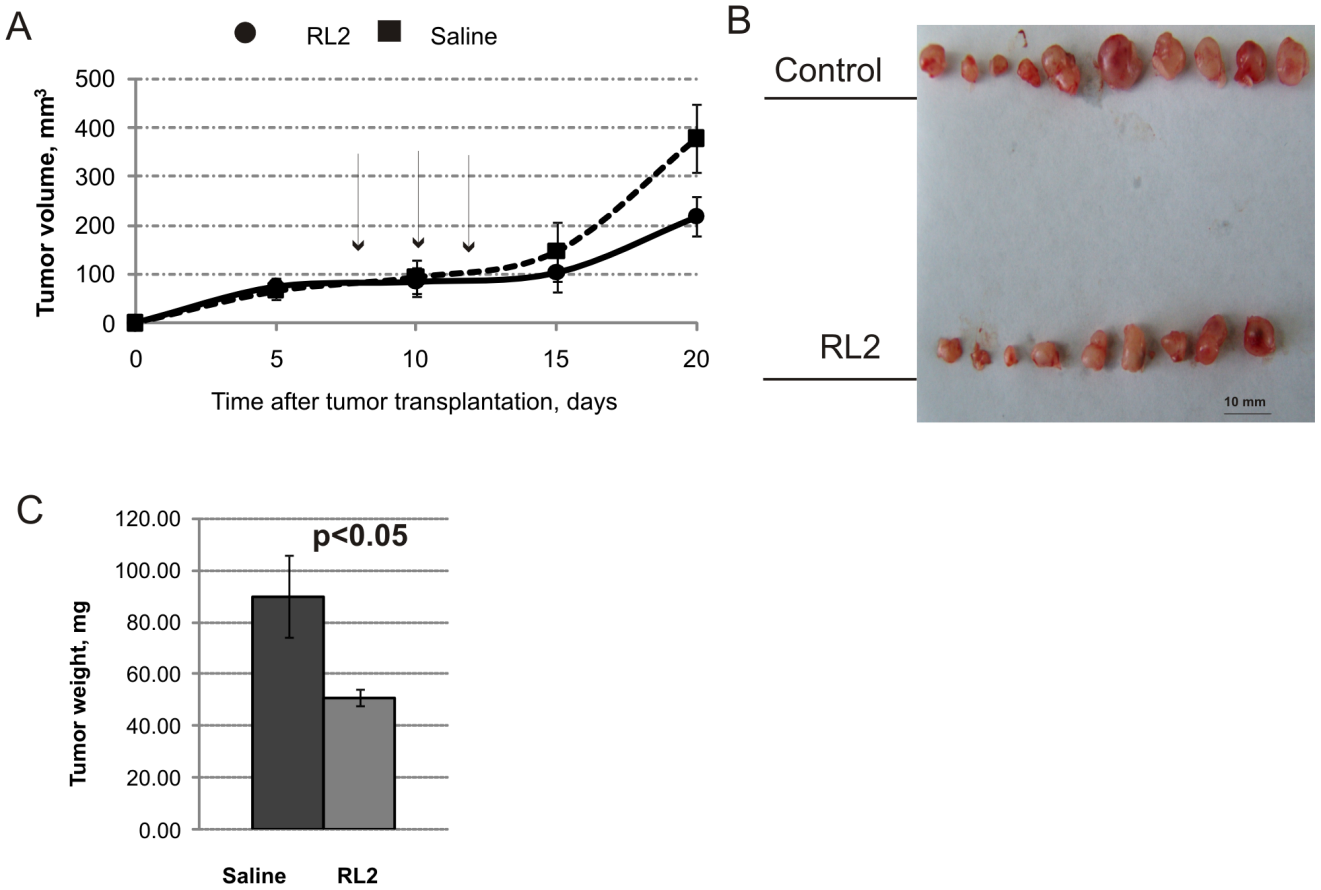
RL2 therapy delays tumour development. Female SCID mice aged 3–4 weeks old were used for s.c. MDA-MB-231 cell transplantation. RL2 (40 mg/kg) dissolved in saline was administered i.v. via the tail vein every second day starting on day 8 after tumour transplantation. **A**. The growth rate of MDA-MB-231 tumours. Arrows indicate the days on which RL2 was injected. **B**: Tumours were excised on day 20 and photographed. **C**. Statistical difference in mean tumour weight between control and experimental groups was p<0.05 (statistically significant differences between groups). Data are presented as mean tumour (g) ± SE.

To test whether the recombinant analogue of lactaptin RL2 delays tumour growth *in vivo*, a mouse xenograft model of breast cancer was used. A suspension of MDA-MB-231 cells in Matrigel was subcutaneously injected into 6–8-week-old female SCID mice to form solid tumours. Tumour volumes were monitored every 5 days. When tumours reached roughly 100 mm^3^, RL2 (40 mg/kg) was injected intravenously every 2 days, with a total of three injections (Fig. 7A). The selected dose of RL2 and the treatment regime were based on a scheme of therapy described previously [5]. Seven days after the last injection the tumours were excised and weighed (Fig. 7B). RL2 injections significantly delayed tumour growth compared to the control group: the average tumour weight was 90±16 mg in the RL2-treated group and 51±3 mg in the control (p<0.05) (Fig. 7C). The rate of inhibition of tumour growth after RL2 therapy was calculated as 43%.

## Discussion

Besides their nutritional function, proteins from human milk have a variety of physiological functions: modulation of the immune system, growth stimulation and defense against pathogens [28], [29]. Natural compounds are considered as a potential basis for the design of new anticancer therapeutics. For instance the tumoricidal activity of HAMLET (human α-lactalbumin made lethal to tumour cells) against bladder cancers *in vivo* was described [30]. However it was later discovered that the protein portion of HAMLET-like complexes play a role only in the delivery of cytotoxic oleic acid molecules into tumour cells across the cell membrane [31]. Lactaptin, the fragment of kappa-casein of human milk, was found to induce the apoptotic death of cultured cancer cells and did not affect non-malignant cells. We assume that lactaptin arises from κ-casein by partial proteolysis. In favour of our hypothesis is the fact that the structure of human κ-casein gene has no alternative splicing sites to produce lactaptin with leader peptide [32]. We suppose that it is unlikely that lactaptin could be directly released by secretory epitheliocytes into milk. The biological function of lactaptin in milk is still unknown, nethertheless human κ-casein proteolytic fragment (63–117) which is a part of lactaptin exhibits cytostatic and cytotoxic effects on *Escherichia coli*, *Staphylococcus carnosus* and some other bacteria [4]. It is known that human κ-casein displays antimicrobial properties inhibiting the adhesion of *Helicobacter pylori* [33]. We therefore speculate that lactaptin can be involved in the ensemble of milk's molecules which provide the protective properties of breastfeeding. In this study the *in vivo* and *in vitro* effects of a recombinant analogue of lactaptin were examined in MDA-MB-231 cells. This cell line was chosen as a model for further *in vivo* research from MCF-7 and SW837 cells after testing the effects of RL2 on cell viability. The breast cancer cell lines MCF-7 and MDA-MB-231 demonstrated greater sensitivity to RL2 than SW837 colon cancer cells. Moreover, in comparison with MDA-MB-231 cells, MCF-7 cells are estrogen-dependent and caspase-3 deficient and we considered these facts to preclude *in vivo* experiments.

In the present study we investigated the mechanisms of RL2-induced cell death and the potential of RL2 as an anticancer drug. Upon treatment with a recombinant analogue of lactaptin we detected apoptosis-typical activation of effector caspase-3 and -7 in the cells. Active caspases -3 and -7 usually cleave the inhibitor of DNase DFF40, and active DFF40 leads to oligonucleosomal fragmentation of DNA and thus results in apoptosis. We observed the appearance of a sub-G1 population of cells with subdiploid DNA content and assumed that RL2 treatment induced apoptosis-typical nuclear fragmentation and cleavage of genomic DNA.

We hypothesized that RL2 probably triggers an intrinsic apoptotic pathway by interacting with intercellular proteins. We attempted to associate RL2-induced cell death with changes in apoptosis-related proteins, including p53, MDM2 and Bcl-2. After 5 h of incubation of MDA-MB-231 cells with RL2 we observed a reduction in the *mdm2* mRNA level and the appearance of a cleaved form of MDM2 protein. The MDM2 protein functions as an E3 ubiquitin ligase that recognizes the N-terminal trans-activation domain (TAD) of the p53 tumour suppressor and as an inhibitor of transcriptional activation of p53. In cancers the function of wild-type p53 is often inhibited by high levels of MDM2, leading to downregulation of tumour suppressive p53 pathways [34]. Destabilization of MDM2 could disturb p53 ubiquitination and lead to further degradation through the ubiquitin-proteasome system that could promote apoptosis. Thus, the drop in *mdm2* mRNA that we observed could restore p53 activity. However we did not detect any changes in p53 mRNA and protein level after RL2 treatment. This observation may indicate that MDM2–p53 interactions involve multiple levels of regulation by numerous cellular proteins and epigenetic mechanisms, meaning that we should not expect a simple correlation between MDM2/p53. Our data provide evidence that RL2 induced p53-independent cell death.

The proteins of the BCL-2 family are the key regulators of apoptosis, exerting both pro- and anti-apoptotic effects [35]. The interactions between apoptosis inhibitors (Bcl-2, Bcl-xL) and inducers (Bax, Bak) belonging to the BCL-2 family and their ratio in the cell in response to multiple death signals determine the cell's fate: death or survival [14], [36]. We have demonstrated in this study that a recombinant analogue of lactaptin RL2 suppressed *bcl-2* mRNA expression and down regulated Bcl-2 protein expression in MDA-MB-231 cells. In the classical model of apoptosis Bcl-2 preserves outer mitochondrial membrane integrity by directly inhibiting the proapoptotic BCL-2 proteins, Bax and Bak, and under apoptotic conditions, a decline in Bcl-2 is accompanied by higher levels of Bax. The ratio of Bax to Bcl-2 was higher at 8 and 24 h in comparison to non-treated cells, consequently apoptotic mechanism could be realized. But it was surprising that after incubation with RL2 the level of Bax was not higher in comparison with non-treated cells. These observations led us to look for other processes involved in RL2-dependent cell death.

As far as binding of the recombinant analogue of lactaptin with cytoskeletal proteins is concerned, we hypothesized that these interactions affect RL2-dependent cell death. The heterodimer of tubulin consists of α/β-tubulin and forms fibers of microtubules. Apoptotic cell death can be induced by antimicrotubule agents that are known to result in the aberrant formation of the mitotic spindle and blockage of the cell cycle in G2/M phase [37]. Microtubule-targeting agents that destabilize (nocodazole, colchicine) or stabilize (paclitaxel, docetaxel) tubulin are the most frequently used anti-cancer drugs [38]. Recent studies showed that microtubule destabilization/stabilization specifically altered mitochondrial ΔΨ [39]. It is thus possible that the induction of mitochondrion membrane permeability in cancer cells by RL2 could be initiated by interactions between RL2 and tubulin [5]. Maldonado et al. demonstrated that free tubulin and protein kinases dynamically regulate mitochondrial function in cancer cells but not in non-transformed primary cells [39]. Thus, despite the fact that RL2 equally effectively penetrates cancer and nonmalignant primary cells, interactions between RL2 and cytoskeleton proteins only result in the death of cancer cells. Alpha-actinin1 is a structural protein of actin filaments that connects actin filaments with integrin receptors to enable cellular force transduction [40]. The interaction between RL2 and α-actinin1 can destabilize complexes of focal adhesion that in turn reduces the activity of focal adhesion kinase (FAK). Under low levels of active FAK the active form of MDM2 ligase decreases, which causes p53 activation and the induction of apoptosis.

Many anticancer substances induce autophagy: this is frequently activated in tumour cells subjected to radiotherapy and chemotherapy and prohibits cell death [41], [42]. During autophagy autophagosomes engulf long-lived proteins, abnormal proteins, protein aggregates and organelles. Since intracellular proteins are degraded by the autophagolysosomal pathway, the authors hypothesize that RL2 could activate autophagy, as well as damaged or misfolded intracellular proteins. Antiapoptotic BCL-2 proteins are involved in the regulation not only of apoptosis but also autophagy [43], and we hypothesize that the reduction of Bcl-2 by RL2 could indicate not only apoptosis but also autophagy. We detected autophagy-like changes in MDA-MB-231 cells by LC3 processing 3 hours after RL2 treatment. This autophagy could be based on the ability of RL2 to penetrate the cells, however this requires more evidence. Whereas autophagy helps rapidly dividing cancer cells to overcome nutrient deficiencies, inhibition of autophagy could increase the sensitivity of tumours to RL2 in the same way as diverse anticancer agents [44], [45]. Chloroquine, the autophagy inhibitor, disrupts lysosomal function and prevents the degradation of protein within the autophagosome. We investigated the effects of a combination of CQ with RL2 on the viability of MDA-MB-231 cells and found that CQ reinforced the effect of RL2. Since a value of CI<1 indicates a synergistic effect of a drug combination we concluded that RL2 and CQ induced cell death in a synergistic fashion [26]. So, on the one hand, we hypothesize that the suppression of autophagolysosome formation by chloroquine promotes RL2 stability in the cells and thus promotes RL2-dependent apoptosis. On the other hand, suppression of autophagolysosome formation could prevent the potential recovery of cells with a small subset of depolarized mitochondrions and result in enhanced cell death [46], [47].

The stable proapoptotic effects of RL2 observed in MDA-MB-231 and MCF-7 cells led us to investigate the antitumour activity of RL2 against tumour cells grafted on immunodeficient mice. MCF-7 tumour xenografts grow well under 17β-estradiol supplementation but estrogen application often induces renal damage and bladder stone formation as side effects and might cause animal death during experiments [48]. We focused our further investigation of the antitumour potential of RL2 on MDA-MB-231 cells that were estrogen-independent in a xenograft model. We demonstrated that the recombinant analogue of lactaptin retarded tumour progression *in vivo* without detectable toxic effects.

In conclusion, this work demonstrates that the recombinant analogue of lactaptin RL2 downregulates Bcl-2 expression and induces p53-independent cell death. We identified α/β-tubulin and α-actinin1 as molecular targets of RL2. Autophagy-related changes were observed during incubation of cells with RL2. The cell death effect was enhanced synergistically when RL2 was combined with the autophagy inhibitor CQ. We have shown that the recombinant analogue of lactaptin is an effective agent suppressing the growth of human adenocarcinoma MDA-MB-231 cells grafted on immunodeficient mice. Our results support further *in vivo* studies of the recombinant analogue of lactaptin as a single anticancer therapeutic as well as a component of combination therapy.
